# Identification of Novel 4′-*O*-Demethyl-epipodophyllotoxin Derivatives as Antitumor Agents Targeting Topoisomerase II

**DOI:** 10.3390/molecules27155029

**Published:** 2022-08-07

**Authors:** Wenli Xi, Hua Sun, Kenneth F. Bastow, Zhiyan Xiao, Kuo-Hsiung Lee

**Affiliations:** 1 Beijing Key Laboratory of Active Substance Discovery and Druggability Evaluation, Institute of Materia Medica, Chinese Academy of Medical Sciences and Peking Union Medical College, Beijing 100050, China; 2 The State Key Laboratory of Bioactive Substance and Function of Natural Medicines, Institute of Material Medica, Chinese Academy of Medical Sciences and Peking Union Medical College, Beijing 100050, China; 3 Natural Products Research Laboratories, UNC Eshelman School of Pharmacy, University of North Carolina, Chapel Hill, NC 27599-7568, USA; 4 Chinese Medicine Research and Development Center, China Medical University and Hospital, Taichung 40402, Taiwan

**Keywords:** 4′-*O*-demethyl-epipodophyllotoxin, topoisomerase II, antitumor agent

## Abstract

C4 variation of 4′-*O*-demethyl-epipodophyllotoxin (DMEP) is an effective approach to optimize the antitumor spectra of this compound class. Accordingly, two series of novel DMEP derivatives were synthesized, and as expected, the antitumor spectra of these derivatives varied with different C4 substituents. Notably, most compounds showed significant inhibition against the etoposide (2)-resistant KBvin cells. Four of the compounds (**11**, **18**, **27** and **28**) induced protein-linked DNA break (PLDB) levels higher than those of GL-331 (**6**) and **2**, and are assumed to be topoisomerase II (topo II) poisons more potent than **6** and **2**. Compound **28**, a potent topo II poison highly effective against KBvin cells, was further evaluated with a panel of tumor cells and was most active against HepG2. This compound also exhibited apparent in vivo antitumor efficacy in hepatoma 22 (H22) mouse model. The results indicated that C4 derivation of DMEP is a feasible approach to identify potent topo II inhibitors with optimized antitumor profiles.

## 1. Introduction

**Figure 1:**
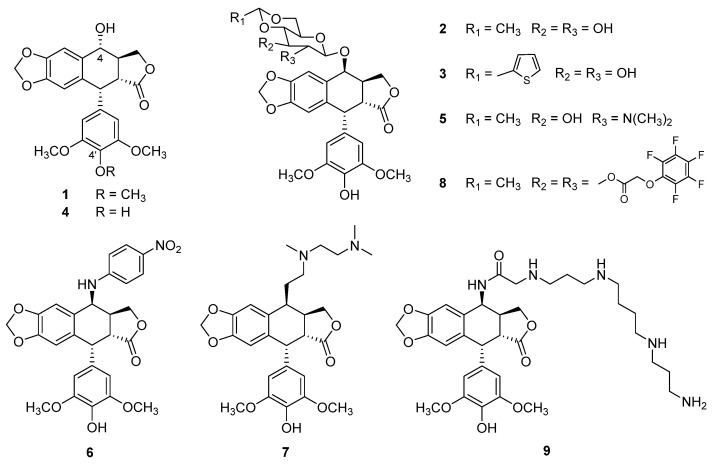
The structures of podophyllotoxin and its representative derivatives.

Lignans are a class of natural products widely distributed in the plant kingdom. Due to their tremendous structural and biological diversity, lignans have been regarded as a precious arsenal for drug discovery [1]. Among them, podophyllotoxin (**1**, Figure 1) is highlighted as an illustrative example to develop therapeutic drugs from bioactive natural lignans. As a cyclolignan abundant in *Podophyllum* species, podophyllotoxin as well as its structurally related lignans exhibit a broad spectrum of biological activities, and antineoplastic and antiviral activities are undoubtedly the most pronounced pharmacological effects. The natural product podophyllotoxin itself is recommended by WHO as a first-line treatment for *Condyloma acuminata*. Furthermore, two semisynthetic derivatives of podophyllotoxin, etoposide (VP-16, **2**) and teniposide (VM-26, **3**), were approved by the FDA as cancer chemotherapy against various cancer types. Recently, VP-16 (**2**) was also trialed for the treatment of cytokine storm in COVID-19 infection [2].

The successful development of these therapeutic drugs has spurred extensive research interest in structural modification of podophyllotoxin to search for potential cancer chemotherapy [2,3]. Although podophyllotoxin itself inhibits microtubulin assembly by acting at the colchicine binding site, alternative molecular mechanisms are involved in the pharmaceutical effects of its derivatives. Different molecular targets, including topoisomerase II (topo II) and insulin-like growth factor 1 receptor, are reported to be responsible for the antineoplastic activities of this compound class [2]. Among the molecular mechanisms involved, topo II inhibition is the primary mechanism for the therapeutic drugs **2** and **3**. Therefore, topo II has been the dominant molecular target for structure–activity relationship (SAR) studies on podophyllotoxin derivatives [4].

Since 4′-demethyl-epipodophyllotoxin (DMEP, **4**) is the fundamental scaffold required for topo II inhibition, DMEP derivation is the major focus for SAR exploration based on the chemical prototype of podophyllotoxin. Intensive efforts have not only revealed conducive SAR clues to guide further structural optimization, but also provided drug candidates for clinical trials. According to currently available data, diverse C4 substituents are generally well accommodated. However, the linking unit immediate to C4 would affect the antitumor spectra of podophyllotoxin derivatives significantly [4]. To overcome the drug resistance and poor water solubility issues associated with compounds **2** and **3**, a variety of substituents were attempted at C4. These endeavors led to the discovery of several drug candidates (**5**–**9**) entering clinical trials [2,4]. NK-611 (**5**) is designed to improve water solubility and shows better bioavailability than that of **2** [5]. GL-331 (**6**) [6] and TOP-53 (**7**) [7] are both topo II inhibitors more potent than **2**, and display unique antitumor spectra. F11782 (**8**) is a dual inhibitor of topoisomerases I and II [8], and F14512 (**9**) targets both topo II and the polyamine transport pathway to facilitate target delivery of the cytotoxic core structure [9].

To fully explore the effects of C4 substituents on the antitumor profiles of podophyllotoxin derivatives, we have previously introduced different C4 substituents to the DMEP scaffold [10,11,12,13,14]. It has been revealed that variation of C4 substituents generally leads to a shift of the antitumor spectrum and represents an effective approach to optimize the antitumor profile of this compound class. Since drug resistance is a common cause of clinical failure in cancer chemotherapy, special attention has been paid to identifying new DMEP derivatives effective against tumor cells resistant to the most frequently used therapeutic drug **2**. A number of new derivatives with potent inhibition against **2**-resistant tumor cells have thereby been identified [10,11]. In particular, compound **XWL-1-48** (**10**) was designed to address both drug resistance and water solubility issues associated with **2**. In the structure of **10**, the anilino moiety immediate to C4 was maintained to modulate the antitumor profile, and a tertiary amino was incorporated as a tail group to increase water solubility. Compound **10** showed potent inhibitory activities against triple-negative breast cancer MDA-MB-231 cells [13] and the multi-drug resistant (MDR) KB subline KBV200 cells [14]. It also turned out to be a topo II inhibitor orally effective in both breast cancer and human hepatocellular carcinoma models [13,14]. Compound **10** was identified as a novel DMEP derivative with unique antitumor profiles and improved water solubility. These efforts further support that C4 derivation of DMEP is a feasible approach to optimize both the pharmacodynamic and pharmacokinetic profiles of this compound class. Accordingly, two series of novel DMEP derivatives with different C4 linkages and bulky tails were designed herein to search for potential antitumor agents with improved pharmacodynamic and/or pharmacokinetic profiles.

## 2. Results and Discussion

### 2.1. Chemistry

**Scheme 1:**
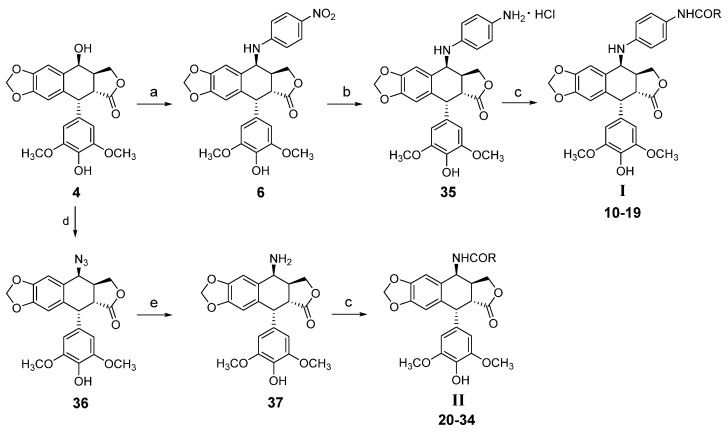
Synthetic routes of target compounds **10**–**34**. Reagents and conditions: (a) MeSO_3_H/NaI, CH_2_Cl_2_, r.t., then *p*-nitroaniline, BaCO_3_, THF; (b) H_2_, 10% Pd-C, EtOAc, pH 1~2, r.t.; (c) corresponding acids, HATU, DIEA, CH_2_Cl_2_, r.t.; (d) CF_3_COOH, NaN_3_, CH_2_Cl_2_, r.t.; (e) H_2_, 10% Pd-C, EtOAc, r.t.

DMEP (**4**) was readily prepared from podophyllotoxin (**1**) according to previously published methods [10], and two series of compounds (**10**–**19** and **20**–**34**) were synthesized as demonstrated in Scheme 1. Briefly, nucleophilic displacement of the hydroxyl in compound **4** by *p*-nitroaniline gave compound **6**, and compound **6** then underwent Pd-C catalyzed hydrogenation to afford intermediate **35**. Subsequent condensation of compound **35** with corresponding acids in the presence of HATU (1-[bis (dimethylamino)methylene]-1*H*-1,2,3-triazolo [4,5-b]pyridinium 3-oxide hexafluorophosphate) and DIEA (*N*,*N*-diisopropylethylamine) provided compounds **10**–**19** in series I. Similarly, nucleophilic displacement of compound **4** with sodium azide gave compound **36**, and Pd-C catalyzed hydrogenation of compound **36** afforded intermediate **37**. Further condensation of **37** with corresponding acids yielded compounds **20**–**34** in series II. All of the compounds were well characterized by physical and spectral data, including melting points, optical rotations, mass and ^1^H-NMR spectra.

### 2.2. Cellular Antitumor Activities of All Target Compounds

All of the target compounds were initially evaluated for their inhibitory activities against several tumor cell lines with the sulforhodamine B (SRB) assay. Etoposide (**2**) is the DMEP-derived therapeutic drug most frequently used in the clinical setting, and GL-331 (**6**) is the clinically investigated DMEP derivative with an anilino moiety immediate to C4. Both were taken as reference compounds (Table 1). According to data listed in Table 1, diverse C4 substituents are generally well accommodated. All of the compounds exhibited significant inhibitory effects on the growth of A549, DU145 and KB cells. However, the antitumor profiles of the derivatives varied with different C4 substituents. Most compounds were generally more effective against A549 or DU145 cells. In contrast, some of the compounds were more potent against KB cells (e.g., **31** and **34**) or even almost equally effective against the three cell lines (e.g., **12** and **19**).

As mentioned earlier, drug resistance is a major cause of clinical failure in cancer chemotherapy. Extensive efforts have been thereby devoted to identifying novel derivatives effective against **2**-resistant tumor cells. KBvin is a multidrug resistant (MDR) KB subline selected with vincristine, and it shows cross-resistance to VP-16 (**2**). GL-331 (**6**) overcomes multidrug resistance in a variety of cancer cell lines, including the **2**-resistant KBvin cells [15]. As shown in Table 1, KBvin cells were obviously resistant to **2**. However, **6** was comparably potent against both KB and KBvin cells and showed a relative resistance of 1.7-fold. Notably, most of the target compounds retained significant inhibitory activity against the **2**-resistant KBvin cells. Several compounds (e.g., **19**, **25**, **29**, **30** and **31**) were even identically potent against KB and KBvin cells. Presumably, these newly obtained compounds shared the superior drug-resistance profile of compound **6**. The results are consistent with previous observation that C4 substituents could affect the antitumor spectrum of podophyllotoxin derivatives significantly. In particular, as observed previously with GL-331 (**6**), an anilino moiety immediate to C4 could result in an improved antitumor profile and overcome multidrug resistance [4,15].

Although multiple factors are responsible for the cellular activity, the cellular data listed in Table 1 still provided some SAR clues. Generally, compounds in series I were more potent than their congeners in series II, which implied the possible existence of a stretched pocket in the active site of topo II to better accommodate the more extended C4 substituents in series I. In addition, compounds lacking a potentially positively charged nitrogen center (e.g., **30**, **31**, **32** and **34**) were generally less potent. This might be attributed to the presence of electrostatic interaction between the positively charged nitrogen center of the derivative and corresponding residues (either negatively charged or aromatic center) in the active site of topo II.

### 2.3. Induction of Protein-Linked DNA Breaks (PLDB) by Selected Compounds

DNA cleavage/ligation reaction is a key step in the catalytic cycle of topo II. Topo II poisons increase levels of topo II–DNA cleavage complexes and convert topo II into a cellular toxin [16]. Etoposide (**2**) is known as a topo II poison, and it effectively blocks transcription and replication by inducing high levels of topo II–DNA covalent complexes [17]. Therefore, induction of protein-linked DNA breaks (PLDB) is considered as a key feature for DMEP derivatives. Accordingly, ten compounds with IC_50_ values better than 6.0 µM and 2.5 µM for the **2**-resistant KBvin cells and the other three kinds of cells, respectively, were selected to further evaluate their capabilities to cause the formation of PLDB, and compound **6** was tested in parallel (Table 2).

The level of PLDB induced by compound **2** was set arbitrarily as 100%, and the levels of PLDB formation resulting from treatment with other compounds were compared with that of **2**. As shown in Table 2, most compounds induced noticeable levels of PLDB. Consistent with the previous observation [6], compound **6** showed a capability significantly superior to **2** in inducing PLDB. Notably, four of the newly synthesized compounds (**11**, **18**, **27** and **28**) induced higher PLDB levels than that of compound **6**, and were postulated to be topo II poisons more potent than compounds **2** and **6**.

Compound **10** was previously identified as a topo II inhibitor more potent than compound **6** by the topo II-mediated kDNA decatenation assay [14]. Surprisingly, it induced a much less impressive level of PLDB as compared to **6**. As a dominant phenotype of topo II poisons, the induction of PLDB is generally considered more specific for topo II poisons, including DMEP derivatives [18]. Consistent with our previous observation [10,11], the induction of PLDB did not correlate with cellular inhibitory effects of the compounds.

### 2.4. Antitumor Effects of Compound ***28***

Among the four compounds inducing higher PLDB levels than compound **6**, compound **28** was most potent against the **2**-resistant KBvin cells and showed the most favorable relative resistance profile (Table 1). Presumably, compound **28** is most favorable to overcome the drug resistance issue associated with **2**. Therefore, it was further assessed against a panel of tumor cells with the MTT assay (Table 3) to explore its therapeutical potential, and both **2** and **6** were tested in parallel.

As revealed by the preliminary evaluation, compound **28** showed an antitumor spectrum apparently broader than those of compounds **2** and **6**. Compound **28** was generally more effective against the tested tumor cell lines than **2**, and it also showed potent inhibition against Bel7402/5-FU, a multi-drug resistant subline insensitive to compounds **2** and **6**. These results further endorsed the critical effects of C4 substituents on the antitumor spectra of podophyllotoxin derivatives.

Compound **28** showed high potency against all three human hepatocellular carcinoma cell lines tested, which suggested a therapeutical potential in the treatment of hepatocellular carcinoma. Therefore, it was evaluated in vivo for its antitumor efficacy in a hepatoma 22 (H22) mouse model. The clinical drug VP-16 (**2**) was tested in parallel as a reference. Toxicity is one of the major concerns with cytotoxic anticancer drugs. Therefore, the changes in the body weight of the experimental animals, which are usually taken as a preliminary indicator of general toxicity, were also monitored. 

When orally administered at 8 mg/kg daily for 11 days, compound **28** could effectively inhibit the tumor growth and decrease tumor weight by 51.0% (Table 4). The **28**-treated mice had a steady gain in body weight and showed no evident difference as compared to the control group, which indicated good tolerance of the experimental animals to compound **28**. In contrast, VP-16 (**2**) decreased the tumor weight by 75.1% when administered at 26 mg/kg daily for 8 days, whereas compound **2** was much less well-tolerated and caused a dramatic decrease in body weight in the mice. The final body weight was even significantly lower than the initial body weight of the mice. Actually, during the experimental period, the administration of compound **2** was discontinued on the day 9, due to the significant decrease in body weight and obvious gross toxicity observed. Therefore, although the tumor growth inhibitory effect of compound **28** seemed to be slightly inferior to compound **2** under the experimental conditions, it might be much less toxic than **2,** as reflected by the alteration in body weight of the mice after treatment. These results suggested that C4 modification of the podophyllotoxin scaffold is a feasible approach to improve not only the antitumor spectrum but also the safety profile.

## 3. Materials and Methods

### 3.1. Chemistry

All melting points were taken on Fisher-Scientific and Mel-Temp II melting point instruments and are uncorrected. ^1^H-NMR spectra were obtained using Varian Mercury 300 NMR spectrometers with TMS as the internal standard. All chemical shifts were reported in ppm. Optical rotations were measured with a Perkin-Elmer 341 LC polarimeter, using CDCl_3_ as solvent. Mass spectra were recorded on an LC/MSD TOF (Agilent Technologies Inc., Santa Clara, CA, USA) instrument equipped with a Turbo Ions Spray ion source. All reagents and solvents were commercially obtained from local suppliers and were used directly without further purification. Natural podophyllotoxin was used for the structural modification and was commercially obtained and purified in-house. HPLC analysis was performed using a Shimadzu LC-20AT system with a YMC-Pack ODS-A (4.6 mm, 250 mm; particle size: 5 µm; pore size: 120 Å) column. The purity of all target compounds was greater than 95%.

**General preparation of compounds 11**–**19.** To a solution of compound **35** (131 mg, 0.25 mmol) in anhydrous CH_2_Cl_2_ (15 mL) were added HATU (105 mg, 0.275 mmol) and appropriate carboxylic acids (0.3 mmol). After 5 min, DIEA (80 mg, 0.61 mmol) was slowly added to the reaction mixture, and the mixture was stirred at ambient temperature overnight. The solvent was washed with saturated NaHCO_3_ and brine, respectively, and the organic layer was then dried over anhydrous sodium sulfate. After the solvent was removed under reduced pressure, the crude product was purified by column chromatography on silica gel. 

Compound **11**: yield: 35.6%; mp: 230–232 °C; [α]_D_^20^: −92.7 (c 0.05, DMF); ^1^H-NMR (300 MHz, DMSO-d_6_): δ 9.66 (s, 1H, -NHCO-), 7.30 (d, 2H, *J* = 8.4 Hz, -ArH), 6.75 (s, 1H, 5-H), 6.66 (d, 2H, *J* = 9.0 Hz, -ArH), 6.53 (s, 1H, 8-H), 6.26 (s, 2H, 2′, 6′-H), 5.98 (d, 2H, *J* = 9.3 Hz, -OCH_2_O-), 5.86 (d, 1H, *J* = 8.4 Hz, 4-H), 4.81 (m, 1H, 11-H), 4.49 (d, 1H, *J* = 5.4 Hz, 1-H), 4.32 (t, 1H, *J* = 7.5 Hz, 11-H), 3.64–3.70 (s, 7H, -NHPh, 3′, 5′-OCH_3_), 3.27–3.28 (m, 1H, 2-H), 2.96–2.99 (m, 1H, 3-H), 2.54–2.57 (m, 2H, -CH_2_CO-), 2.35–2.40 (m, 2H, -CH_2_-), 2.18 (s, 6H, -N(CH_3_)_2_); MS (*m/z*): 645 [M+H]^+^.

Compound **12**: yield: 50.9%; mp: 192–194 °C; [α]_D_^20^: −82 (c 0.05, CHCl_3_); ^1^H-NMR (300 MHz, CDCl_3_): δ 7.45 (d, 2H, *J* = 7.2 Hz, -ArH), 7.15 (s, 1H, -NHCO-), 6.74 (s, 1H, 5-H), 6.49–6.52 (m, 3H, -ArH), 6.33 (s, 2H, 2′, 6′-H), 5.96 (d, 2H, *J* = 4.5 Hz, -OCH_2_O-), 4.59–4.63 (m, 2H, 1-H, 4-H), 4.35 (t, 1H, *J* = 7.8 Hz, 11-H), 3.98 (t, 1H, *J* = 9.3 Hz, 11-H), 3.79 (s, 7H, -NHPh, 3′, 5′-OCH_3_), 3.14 (dd, 1H, *J* = 4.8, 10.2 Hz, 2-H), 2.93–2.97 (m, 2H, -CH_2_, 3-H), 2.31 (s, 3H, -CH_3_), 1.89–2.18 (m, 9H, piperidinyl-H); MS (*m/z*): 616 [M+H]^+^.

Compound **13**: yield: 30.6%; mp: 119–122 °C; [α]_D_^20^: −94 (c 0.05, DMF); ^1^H-NMR (300 MHz, CDCl_3_): δ 10.99 (s, 1H, -NHCO-), 7.39 (d, *J* = 8.4 Hz, 2H, -ArH), 6.75 (s, 1H, 5-H), 6.51–6.53 (m, 3H, -ArH), 6.33 (s, 2H, 2′, 6′-H), 5.95 (d, 2H, *J* = 5.4 Hz, -OCH_2_O-), 4.63 (d, 1H, *J* = 3.9 Hz, 4-H), 4.58 (d, 1H, *J* = 4.8 Hz, 1-H), 4.39 (t, 1H, *J* = 8.4 Hz, 11-H), 4.01 (t, 1H, *J* = 10.2 Hz, 11-H), 3.83 (s, 6H, 3′, 5′-OCH_3_), 3.16 (dd, 1H, *J* = 5.1, 14.1 Hz, 2-H), 2.97–3.03 (m, 1H, 3-H), 1.68–2.72 (m, 14H, -CH_2_CH_2_-piperidinyl-H); MS (*m/z*): 630 [M+H]^+^.

Compound **14**: yield: 38.2%; mp: 166–167 °C; [α]_D_^20^: −117 (c 0.05, CHCl_3_); ^1^H-NMR (300 MHz, DMSO-*d_6_*): δ 10.51 (s, 1H, -NHCO-), 7.38 (d, 2H, *J* = 8.4 Hz, -ArH), 6.74 (s, 1H, 5-H), 6.51–6.53 (m, 3H, -ArH), 6.34 (s, 2H, 2′, 6′-H), 5.97 (d, 2H, *J* = 4.2 Hz, -OCH_2_O-), 5.44 (s, 1H, -OH), 4.64 (t, 1H, *J* = 5.1 Hz, 4-H), 4.60 (d, 1H, *J* = 4.8 Hz, 1-H), 4.39 (t, 1H, *J* = 8.1 Hz, 11-H), 4.01 (t, 1H, *J* = 10.5 Hz, 11-H), 3.75–3.81 (m, 10H, 3′, 5′-OCH_3_, morpholinyl-H), 3.16 (dd, 1H, *J* = 5.1, 14.1 Hz, 2-H), 2.97–3.05 (m, 1H, 3-H), 2.75 (t, 2H, *J* = 5.7 Hz, -COCH_2_CH_2_N-), 2.62 (s, 4H, morpholinyl-H), 2.54 (t, 2H, *J* = 5.7 Hz, -COCH_2_CH_2_N-); MS (*m/z*): 632 [M+H]^+^.

Compound **15**: yield: 44.7%; mp: 159–161 °C; [α]_D_^20^: −123 (c 0.05, DMF); ^1^H-NMR (300 MHz, CDCl_3_): δ 7.39 (d, 2H, *J* = 8.4 Hz, -ArH), 6.74 (s, 1H, 5-H), 6.50–6.52 (m, 3H, -ArH), 6.33 (s, 2H, 2′,6′-H), 5.96 (d, 2H, *J* = 3.0 Hz, -OCH_2_O-), 5.52 (s, 1H, -OH), 4.58–4.63 (m, 2H, 1-H, 4-H), 4.38 (t, 1H, *J* = 7.5 Hz, 11-H), 4.02 (t, 1H, *J* = 9.6 Hz, 11-H), 3.79 (s, 6H, 3′, 5′-OCH_3_), 3.75 (s, 1H, -NHPh), 3.16 (dd, 1H, *J* = 5.1, 14.1 Hz, 2-H), 3.01–3.09 (m, 1H, 3-H), 2.52–2.74 (m, 12H, -CH_2_CH_2_-piperazidinyl-H,), 2.36 (s, 3H, N-CH_3_); MS (*m/z*): 645 [M+H]^+^.

Compound **16**: yield: 31.2%; mp: 177–178 °C; [α]_D_^20^: −78 (c 0.05, CHCl_3_); ^1^H-NMR (300 MHz, CDCl_3_): δ 7.20 (m, 7H, ArH,), 7.11 (s, 1H, -NHCO-), 6.73 (s, 1H, 5-H), 6.50–6.52 (m, 3H, -ArH), 6.32 (s, 2H, 2′, 6′-H), 5.96 (s, 2H, -OCH_2_O-), 4.58–4.60 (m, 2H, 1-H, 4-H), 4.35 (t, 1H, *J* = 8.4 Hz, 11-H), 3.97 (t, 1H, *J* = 10.2 Hz, 11-H), 3.79 (s, 6H, 3′, 5′-OCH_3_), 3.54 (s, 2H, -CH_2_Ph), 2.97–3.17 (m, 4H, 2-H, 3-H, piperidinyl-H), 1.67–2.32 (m, 7H, piperidinyl-H); MS (*m/z*): 692 [M+H]^+^.

Compound **17**: yield: 43.5%; mp: 162–163 °C; [α]_D_^20^: −57 (c 0.1, CHCl_3_); ^1^H-NMR (300 MHz, DMSO-*d_6_*): δ 9.47 (s, 1H, -NHCO-), 8.27 (s, 1H, -OH), 8.19 (d, 2H, *J* = 8.1 Hz, -ArH), 7.60 (d, 2H, *J* = 8.4 Hz, -ArH), 7.31 (d, 2H, *J* = 8.4 Hz, -ArH), 6.74 (s, 1H, 5-H), 6.62 (d, 2H, *J* = 8.7 Hz, -ArH), 6.52 (s, 1H, 8-H), 6.25 (s, 2H, 2′, 6′-H), 5.97 (d, 2H, *J* = 9.3 Hz, -OCH_2_O-), 5.85 (d, 1H, *J* = 8.4 Hz, 4-H), 4.80 (s, 1H, 11-H), 4.48 (d, 1H, *J* = 4.5 Hz, 1-H), 4.32 (t, *J* = 6.9 Hz, 1H, 11-H), 3.63–3.72 (m, 7H, -NHPh, 3′, 5′-OCH_3_), 3.60 (s, 1H, -COCH-), 3.25–3.27 (m, 1H, 2-H), 2.90–3.07 (m, 1H, 3-H), 2.84 (d, 2H, *J* = 9.9 Hz, -CH_2_Ph), 1.64–2.25 (m, 8H, piperidinyl-H); MS (*m/z*): 737 [M+H]^+^.

Compound **18**: yield: 56.7%; mp: 126–128 °C; [α]_D_^20^: −141 (c 0.05, CHCl_3_); ^1^H-NMR (300 MHz, DMSO-*d_6_*): δ 9.65 (s, 1H, -OH), 8.85 (s, 1H, -NHCO-), 7.29–8.27 (m, 7H, -ArH), 6.75 (s, 1H, 5-H), 6.63 (d, 2H, *J* = 8.1 Hz, -ArH), 6.53 (s, 1H, 8-H), 6.25 (s, 2H, 2′, 6′-H), 5.97 (d, 2H, *J* = 9.6 Hz, -OCH_2_O-), 5.86 (d, 1H, *J* = 8.1 Hz, 4-H), 4.80–4.82 (m, 1H, 11-H), 4.49 (d, 1H, *J* = 4.5 Hz, 1-H), 4.33 (s, 1H, 11-H), 3.63–3.73 (m, 7H, -NHPh, 3′, 5′-OCH_3_), 3.50 (s, 2H, -COCH_2_CH_2_N-), 3.03 (s, 4H, 2, 3-H, -NCH_2_Ph), 2.67 (s, 2H, -COCH_2_CH_2_N-), 2.14 (s, 3H, -NCH_3_); MS (*m/z*): 666 [M+H]^+^. 

Compound **19**: yield: 26.3%; mp: 178–180 °C; [α]_D_^20^: −148 (c 0.05, DMF); ^1^H-NMR (300 MHz, CDCl_3_): δ 7.57 (d, 2H, *J* = 8.7 Hz, -ArH), 7.61 (s, 1H, -NHCO-), 7.45 (d, 2H, *J* = 8.4 Hz, -ArH), 6.69–6.77 (m, 3H, -ArH), 6.50–6.56 (m, 3H, -ArH), 6.33 (s, 2H, 2′, 6′-H), 5.96 (d, 2H, *J* = 6.0 Hz, -OCH_2_O-), 5.30 (s, 1H, -OH), 4.57–4.65 (m, 2H, 1-H, 4-H), 4.39 (t, 1H, *J* = 7.5 Hz, 11-H), 4.02 (t, 1H, *J* = 10.2 Hz, 11-H), 3.78 (s, 6H, 3′, 5′-OCH_3_), 3.16 (dd, 1H, *J* = 4.8, 14.1 Hz, 2-H), 3.04 (s, 6H, -N(CH_3_)_2_), 2.88–3.01 (m, 1H, 3-H); MS (*m/z*): 638 [M+H]^+^.

**Generalpreparation of compounds 20**–**34.** To a solution of compound **37** (100 mg, 0.25 mmol) in anhydrous CH_2_Cl_2_ (15 mL) were added HATU (105 mg, 0.275 mmol) and appropriate carboxylic acids (0.3 mmol). After 5 min, DIEA (0.61 mmol) was slowly added to the reaction mixture, and the mixture was stirred at ambient temperature overnight. The solvent was washed with saturated NaHCO_3_ and brine, respectively. The organic layer then was dried over anhydrous sodium sulfate. After the solvent was removed under reduced pressure, the crude product was purified by column chromatography on silica gel.

Compound **20**: yield: 75.8%; mp: 145–148 °C; [α]^20^: −94 (c 0.05; CDCl_3_); ^1^H-NMR (300 MHz, CDCl_3_): δ 8.75 (d, *J* = 7.2 Hz, 1H, -NH-CO-), 6.76 (s, 1H, 5-H), 6.51 (s, 1H, 8-H), 6.29 (s, 2H, 2′, 6′-H), 5.97 (s, 2H, -OCH_2_O-), 5.22 (d, *J* = 5.4 Hz, 1H, 4-H), 4.59 (d, *J* = 4.2 Hz, 1H, 1-H), 4.42 (t, *J* = 8.1 Hz, 1H, 11-H), 3.86 (t, *J* = 9.9 Hz, 1H, 11-H), 3.77 (s, 6H, 3′,5′-OCH_3_), 2.85–2.92 (m, 1H, 3-H), 2.78 (dd, *J* = 4.8 Hz, 14.1 Hz, 1H, 2-H), 2.44–2.55 (m, 2H, -CO-CH_2_-), 2.39–2.41 (m, 2H, -CH_2_-N(CH_3_)_2_), 2.16 (s, 6H, -N(CH_3_)_2_); MS (*m/z*): 499 [M+H]^+^.

Compound **21**: yield: 55.7%; mp: 159–161 °C; [α]^20^: −105 (c 0.05; CDCl_3_); ^1^H-NMR (300 MHz, CDCl_3_): δ 6.69 (s, 1H, 5-H), 6.50 (s, 1H, 8-H), 6.26 (s, 2H, 2′,6′-H), 5.96 (d, *J* = 4.8 Hz, 2H, -OCH_2_O-), 5.75 (d, *J* = 6.3 Hz, 1H, -NH-CO-), 5.18 (q, 1H, 11-H), 4.53 (d, *J* = 4.2 Hz, 1H, 1-H), 4.39 (t, *J* = 7.5 Hz, 1H, 11-H), 3.74–3.76 (m, 7H, 3′,5′-OCH_3_, 4-H), 2.78–2.90 (m, 4H, piperidinyl-H, 2,3-H), 2.25 (s, 3H, -CH_3_), 1.75–2.16 (m, 7H, piperidinyl-H); MS (*m/z*): 525 [M+H]^+^.

Compound **22**: yield: 78.2%; mp: 134–137 °C; [α]^20^: −61 (c 0.05; CDCl_3_); ^1^H-NMR (300 MHz, CDCl_3_): δ 9.48 (s, 1H, -NH-CO-), 6.78 (s, 1H, 5-H), 6.53 (s, 1H, 8-H), 6.31 (s, 2H, 2′,6′-H), 5.96 (d, *J* = 14.1 Hz, 2H, -OCH_2_-), 5.18 (d, *J* = 4.8 Hz, 1H, 4-H), 4.62 (d, *J* = 4.5 Hz, 1H, 1-H), 4.41 (t, *J* = 4.1 Hz, 1H, 11-H), 3.83 (t, *J* = 10.2 Hz, 1H, 11-H), 3.78 (s, 6H, 3′, 5′-OCH_3_), 2.86–2.88 (m, 2H, 2-H, 3-H), 2.81 (s, 2H, -CO-CH_2_-CH_2_-N-), 2.61 (s, 2H, -CO-CH_2_-CH_2_-N-), 1.25–2.42 (m, 10H, piperidinyl-H); MS (*m/z*)**:** 539 [M+H]^+^.

Compound **23**: yield: 77%; mp: 121–122 °C; [α]^20^: −69 (c 0.05; CDCl_3_); ^1^H-NMR (300 MHz, CDCl_3_): δ 8.81 (s, 1H, -NH-CO-), 6.75 (s, 1H, 5-H), 6.55 (s, 1H, 8-H), 6.30 (s, 2H, 2′,6′-H), 5.96 (d, *J* = 7.5 Hz, 2H, -OCH_2_O-), 5.45 (s, 1H, -OH), 5.18 (t, *J* = 4.8 Hz, 1H, 4-H), 4.61 (d, *J* = 4.8 Hz, 1H, 1-H), 4.43 (t, *J* = 8.1 Hz, 1H, 11-H), 3.89 (t, *J* = 10.2 Hz, 1H, 11-H), 3.78 (s, 6H, 3′,5′-OCH_3_), 3.27–3.41 (m, 4H, -CO-CH_2_-CH_2_-N-, morpholinyl-H), 2.90–2.98 (m, 1H, 3-H), 2.87 (dd, *J* = 4.8 Hz, 14.4 Hz, 1H, 2-H), 2.65 (t, *J* = 5.4 Hz, 2H, -CO-CH_2_-CH_2_-N-), 2.39–2.43 (m, 6H, morpholinyl-H); MS (*m/z*)**:** 541 [M+H]^+^.

Compound **24**: yield: 52.2%; mp: 211–213 °C; [α]^20^: −101 (c 0.05; CDCl_3_); ^1^H-NMR (300 MHz, CDCl_3_): δ 9.26 (s, 1H, -NH-CO-), 6.76 (s, 1H, 5-H), 6.55 (s, 1H, 8-H), 6.30 (s, 2H, 2′,6′-H), 5.96 (d, *J* = 6.9 Hz, 2H, -OCH_2_O-), 5.47 (s, 1H, -OH), 5.14–5.18 (m, 1H, 4-H), 4.62 (d, *J* = 5.1 Hz, 2H, 1-H), 4.43 (t, *J* = 8.4 Hz, 1H, 11-H), 3.89 (t, *J* = 9.9 Hz, 1H, 11-H), 3.78 (s, 6H, 3′,5′-OCH_3_), 2.93–2.98 (m, 1H, 3-H), 2.83 (dd, *J* = 5.1 Hz, 14.4 Hz, 1H, 2-H), 2.39–2.66 (m, 12H, -CH_2_-CH_2_-piperazidinyl-H), 2.14 (s, 3H, N-CH_3_); MS (*m/z*): 554 [M+H]^+^.

Compound **25**: yield: 73.3%; mp: 112–114 °C; [α]^20^: −72 (c 0.05; CDCl_3_); ^1^H-NMR (300 MHz, CDCl_3_): δ 7.28–7.32 (m, 5H, -ArH), 6.70 (s, 1H, 5-H), 6.51 (s, 1H, 8-H), 6.28 (s, 2H, 2′,6′-H), 5.98 (d, *J* = 4.8 Hz, 2H, -OCH_2_-), 5.68 (d, *J* = 6.6 Hz, 1H, 4-H), 5.20 (t, *J* = 4.1 Hz, 1H, 11-H), 4.57 (d, *J* = 4.2 Hz, 1H, 1-H), 4.45 (t, *J* = 8.7 Hz, 1H, 11-H), 3.77 (s, 6H, 3′,5′-OCH_3_), 3.52 (s, 2H, -CH_2_-), 2.84–2.98 (m, 3H, 2-H, 3-H, -CO-CH-), 1.76–2.19 (m, 8H, piperidinyl-H); MS (*m/z*)**:** 601 [M+H]^+^.

Compound **26**: yield: 43.5%; mp: 244 °C; [α]^20^: −52.7 (c 0.05; CDCl_3_); ^1^H-NMR (300 MHz, CDCl_3_): δ 8.16 (d, *J* = 8.7 Hz, 2H, -ArH), 7.50 (d, *J* = 8.4 Hz, 2H, -ArH), 6.71 (s, 1H, 5-H), 6.52 (s, 1H, 8-H), 6.28 (s, 2H, 2′,6′-H), 5.98 (d, *J* = 5.4 Hz, 2H, -OCH_2_O-), 5.63 (d, *J* = 6.9 Hz, 1H, -NH-CO-), 5.22 (t, 1H, *J* = 4.5 Hz, 11-H), 4.58 (d, *J* = 4.8 Hz, 1H, 1-H), 4.42 (t, *J* = 7.8 Hz, 1H, 11-H), 3.72–3.77 (m, 7H, 4-H, 3′,5′-OCH_3_), 3.57 (s, 2H, -N-CH_2_-Ph), 2.83–2.99 (m, 4H, 2-H, 3-H, piperidinyl-H), 1.79–2.17 (m, 7H, piperidinyl-H); MS (*m/z*)**:** 646 [M+H]^+^.

Compound **27**: yield: 74%; mp: 149–152 °C; [α]^20^: −112.7 (c 0.05; CDCl_3_); ^1^H-NMR (300 MHz, CDCl_3_): δ 7.25 (t, *J* = 8.4, 2H, ArH), 7.00 (t, *J* = 8.4 Hz, 2H, -ArH), 6.70 (s, 1H, 5-H), 6.52 (s, 1H, 8-H), 6.28 (s, 2H, 2′,6′-H), 5.97 (d, *J* = 5.7 Hz, 2H, -OCH_2_O-), 5.64 (d, *J* = 5.7 Hz, 1H, -NH-CO-), 5.45 (s, 1H, -OH), 5.18–5.22 (m, 1H, 11-H), 4.57 (d, *J* = 4.5 Hz, 1H, 1-H), 4.41 (t, *J* = 8.7 Hz, 1H, 11-H), 3.71–3.77 (m, 7H, 4-H, 3′,5′-OCH_3_), 3.44 (s, 2H, -NCH_2_-Ph), 2.89–2.96 (m, 3H, 3-H, piperidinyl-H), 2.81 (dd, *J* = 4.5 Hz, 14.1 Hz, 1H, 2-H), 1.74–2.17 (m, 7H, piperidinyl-H); MS (*m/z*): 619 [M+ H]^+^.

Compound **28**: yield: 86%; mp: 104–105 °C; [α]^20^: −105.3 (c 0.05; CDCl_3_); ^1^H-NMR (300 MHz, DMSO-d_6_): δ 8.39 (d, *J* = 8.4 Hz, 1H, -NH-CO-), 8.25 (s, 1H, -OH), 7.19–7.29 (m, 5H, ArH), 6.77 (s, 1H, 5-H), 6.54 (s, 1H, 8-H), 6.28 (s, 2H, 2′,6′-H), 5.98 (d, *J* = 10.2 Hz, 2H, -OCH_2_O-), 5.15–5.19 (m, 1H, 4-H), 4.50 (d, *J* = 4.8 Hz, 1H, 1-H), 4.28 (m, 1H, 11-H), 3.88 (m, 1H, 11-H), 3.63 (s, 6H, 3′,5′-OCH_3_), 3.45 (q, 2H, -CH_2_-CH_2_-N-CH_2_-Ph), 3.10 (dd, *J* = 4.8 Hz, 14.1, 1H, 2-H), 2.92–2.99 (m, 1H, 3-H), 2.25–2.71 (m, 4H, -CH_2_-CH_2_-N-CH_2_-Ph), 2.05 (s, 3H, -N-CH_3_); MS (*m/z*): 575 [M+H]^+^.

Compound **29**: yield: 58.8%; mp: 169–171 °C; [α]^20^: −94 (c 0.05; CDCl_3_); ^1^H-NMR (300 MHz, CDCl_3_): δ 7.66 (d, *J* = 8.7 Hz, 2H, -ArH), 6.82 (s, 1H, 5-H), 6.65 (d, *J* = 8.7 Hz, 2H, -ArH), 6.55 (s, 1H, 8-H), 6.32 (s, 2H, 2′,6′-H), 5.97 (d, *J* = 5.1 Hz, 2H, -OCH_2_O-), 5.20 (q, 1H, 11-H), 4.61 (d, *J* = 4.5 Hz, 1H, 1-H), 4.41 (t, *J* = 7.5 Hz, 1H, 4-H), 3.95 (t, *J* = 10.2 Hz, 1H, 11-H), 3.77 (s, 6H, 3′,5′-OCH_3_), 2.96–3.03 (m, 8H, 2-H, 3-H, -N(CH_3_)_2_); MS (*m/z*)**:** 547 [M+H]^+^.

Compound **30**: yield: 75.3%; mp: 219–220 °C; [α]^20^: −82 (c 0.05; CDCl_3_); ^1^H-NMR (300 MHz, CDCl_3_): δ 7.70 (d, *J* = 8.4 Hz, 2H, -ArH), 7.29 (d, *J* = 8.4 Hz, 2H, -ArH), 6.80 (s, 1H, 5-H), 6.56 (s, 1H, 8-H), 6.32 (s, 2H, 2′,6′-H), 6.23 (d, *J* = 6.6 Hz, 1H, -NH-CO-), 5.98 (d, *J* = 5.1 Hz, 2H, -OCH_2_O-), 5.40–5.44 (m, 2H, -OH, 4-H), 4.62 (d, *J* = 4.8 Hz, 1H, 11-H), 4.50 (q, 1H, 11-H), 3.91 (t, *J* = 10.2 Hz, 1H, 11-H), 3.79 (s, 6H, 3′,5′-OCH_3_), 2.97–3.06 (m, 1H, 3-H), 2.93 (dd, *J* = 4.8 Hz, 14.1 Hz, 1H, 2-H), 1.25–2.38 (m, 11H, cyclohexyl-H); MS (*m/z*)**:** 586 [M+H]^+^.

Compound **31**: yield: 58.4%; mp: 133–136 °C; [α]^20^: −69 (c 0.05; CDCl_3_); ^1^H-NMR (300 MHz, CDCl_3_): δ 7.15 (d, *J* = 8.4 Hz, 2H, -ArH), 6.87 (d, *J* = 8.7 Hz, 2H, -ArH), 6.65 (s, 1H, 5-H), 6.47 (s, 1H, 8-H), 6.25 (s, 2H, 2′,6′-H), 5.96 (d, *J* = 2.4 Hz, 2H, -OCH_2_O-), 5.60 (d, *J* = 7.2 Hz, 1H, -NH-CO-), 5.29 (s, 1H, -OH), 5.20 (q, 1H, 11-H), 4.49 (d, *J* = 5.1 Hz, 1H, 1-H), 4.38 (t, *J* = 8.4 Hz, 1H, 11-H), 3.67–3.80 (m, 9H, 3′, 5′-OCH_3_, -CH_2_-Ph, 4-H), 3.61(s, 3H, Ph-OCH_3_), 2.84–2.91 (m, 1H, 3-H), 2.63 (dd, *J* = 4.8 Hz, 14.1 Hz, 1H, 2-H); MS (*m/z*)**:** 548 [M+H]^+^.

Compound **32**: yield: 70%; mp: 184–186 °C; [α]^20^: −97 (c 0.05; CDCl_3_); ^1^H-NMR (300 MHz, CDCl_3_): δ 8.26 (s, 1H, -NH-Ph), 7.52 (d, *J* = 7.8 Hz, 1H, -ArH), 7.38 (d, *J* = 7.8 Hz, 1H, -ArH), 7.14–7.23 (m, 3H, -ArH, -C=CH-), 6.57 (s, 1H, 5-H), 6.38 (s, 1H, 8-H), 6.22 (s, 2H, 2′, 6′-H), 5.91 (d, *J* = 9.9 Hz, 2H, OCH_2_O), 5.78 (d, *J* = 7.5 Hz, 1H, -NH-CO-), 5.40 (s, 1H, -OH), 5.18–5.22 (m, 1H, 11-H), 4.37–4.43 (m, 2H, 1-H, 11-H), 3.74–3.83 (m, 9H, 3′,5′-OCH_3_, -CO-CH_2_-, 4-H), 2.80–2.89 (m, 1H, 3-H), 2.46 (dd, *J* = 5.1 Hz, 14.4 Hz, 1H, 2-H); MS (*m/z*)**:** 557 [M+H]^+^, 579 [M+Na]^+^.

Compound **33**: yield: 32.4%; mp: 176–178 °C; [α]^20^: −84 (c 0.05; CDCl_3_); ^1^H-NMR (300 MHz, CDCl_3_): δ 7.54 (d, *J* = 7.5 Hz, 1H, -NH-CO-), 7.07–7.21 (m, 4H, -ArH), 6.72 (s, 1H, 5-H), 6.51 (s, 1H, 8-H), 6.28 (s, 2H, 2′, 6′-H), 5.98 (d, *J* = 1.5 Hz, 2H, -OCH_2_O-), 5.13 (dd, *J* = 4.5 Hz, 6.9 Hz, 1H, 11-H), 4.53 (d, *J* = 4.8 Hz, 1H, 1-H), 4.23 (t, *J* = 8.4 Hz, 1H, 11-H), 3.90–4.04 (m, 2H, 4-H, -CO-CH-NH-), 3.76 (s, 6H, 3′,5′-OCH_3_), 2.08–3.30 (m, 5H, 3-H, -CH_2_- X 2), 2.53 (dd, *J* = 5.1 Hz, 14.1 Hz, 1H, 2-H); MS (*m/z*)**:** 559 [M+H]^+^.

Compound **34**: yield: 78%; mp: 187–189 °C; [α]^20^: −59 (c 0.05; CDCl_3_); ^1^H-NMR (300 MHz, DMSO-d_6_): δ 8.44 (d, *J* = 8.1 Hz, 1H, -NH-CO-), 8.27 (s, 1H, -OH), 7.84 (s, 1H, -NH-CO-), 6.80 (s, 1H, 5-H), 6.55 (s, 1H, 8-H), 6.25 (s, 2H, 2′, 6′-H), 6.00 (d, *J* = 8.1 Hz, 2H, -OCH_2_O-), 5.13 (m, 1H, 11-H), 4.50 (d, *J* = 5.1 Hz, 1H, 1-H), 4.29 (t, *J* = 7.8 Hz, 1H, 4-H), 4.02 (t, *J* = 3.3 Hz, 1H, -NH-CH-), 3.78 (t, *J* = 10.5 Hz, 1H, 11-H), 3.63 (s, 6H, 3′,5′-OCH_3_), 3.21 (dd, *J* = 5.1 Hz, 14.1 Hz, 1H, 2-H), 2.89–2.98 (m, 1H, 3-H), 1.98–2.26 (m, 4H, -CH_2_-CH_2_-CO-); MS (*m/z*)**:** 511 [M+H]^+^, 533 [M+Na]^+^, 1021 [2M+H]^+^.

### 3.2. Biology

**Cell growth inhibition assay**. Cell growth inhibition was measured by following the sulforhodamine B (SRB) protocol developed by Rubinstein et al. [19]. The protein dye SRB binds to protein basic amino acid residues in a pH-dependent way. Generally, it binds to the residues under mild acidic conditions and is solubilized under mild basic conditions for measurement. Briefly, drug stock solutions with final solvent concentrations of less than 2% were prepared in DMSO, and the tumor cells were cultured at 37 °C with 25 mM N-2-hydroxyethylpiperazine-N′-2-ethanesulfonic acid (HEPES), 2% sodium bicarbonate, 10% fetal bovine serum, and 100 µg/mL kanamycin in a humidified atmosphere containing 5% CO_2_. After drug exposure for 72 h, the cells were fixed with trichloroacetic acid (TCA) and stained by SRB. The dye was then solubilized, and the GI_50_ value was interpolated from dose–response data. 

**Cellular protein–DNA complex formation assay**. Induction of intracellular PLDB level was determined by a standard assay method [20]. Briefly, KB cells were labeled with [methyl-^3^H]-thymidine, incubated for 2 h, and then treated with test compounds at 5 µg/mL. After 1 h, the PLDB levels were measured as potassium-SDS precipitable radioactivity.

**Cell viability assay.** Cell viability was determined by MTT assay [21]. Briefly, cells were seeded in 96-well plates and then incubated at 37 °C in a humidified atmosphere containing 5% CO_2_ overnight. The test compounds at various concentrations were added, and the cells were incubated for an additional 72 h. One hundred microliters of 0.5 µg/mL MTT was then added into each well and incubated for another 4h. IC_50_ was measured by quantitating the resulting formazan at 570 nm using a micro-plate reader (Bio-Rad Model 450). 

**In vivo efficacy in H22 mouse model**. All animal experiments were approved by the Ethics Committee for Animal Experiments of the Institute of Materia Medica, Chinese Academy of Medical Sciences and Peking Union Medical College and conducted in accordance with the Guidelines for Animal Experiments of Peking Union Medical College. The assay was performed as follows. H22 mouse model was established in male ICR mice (Center of Experimental Animals, Chinese Academy of Medical Sciences) with body weight of 22–25 g. Ascites containing H22 cells were extracted under sterile conditions and diluted with sterile saline to an appropriate concentration. Each mouse was inoculated subcutaneously with 0.2 mL of tumor fluid in the upper right axilla. The mice were randomly divided into four groups with 10 mice in each group. The control group was only treated with sterile normal saline. The **2**-treated group was orally administered VP-16 with a dose of 26 mg/kg for eight consecutive days, whereas the **28**-treated group was orally administered **28** with a dose of 8 mg/kg for eleven consecutive days. Tumors were excised from the mice and weighed, and the TGI% was calculated. The body weights of mice in all of the groups were also recorded on the 12th day.

## 4. Conclusions

To improve the antitumor profile of podophyllotoxin analogs, two series of novel DMEP derivatives with different C4 substituents were designed and synthesized. Compounds more cellularly active than VP-16 (**2**) and GL-331 (**6**) were identified. Four of them induced high levels of PLDB and were postulated to be topo II poisons more potent than compounds **2** and **6**. Compound **28** demonstrated an antitumor spectrum broader than that of compound **6**. It also displayed significant in vivo antitumor effect in an H22 mouse model without obvious effects on the body weight of the mice. Further safety profiling of compound **28** should be performed. Notably, the results indicated that compound **28** has an antitumor profile superior to that of the previously clinically investigated GL-331 (**6**). It was not only more active against selected tumor cell lines, but also more potent in the PLDB induction assay. The results suggested that C4 derivation of DMEP could be a practical approach to the discovery of novel derivatives as potent topo II inhibitors with improved antitumor profiles.

## 5. Patents

A Chinese patent (ZL201010622267.6) resulting from part of the work reported in this manuscript was granted in 2015.

## Figures and Tables

**Table 1:** Growth inhibitory effects of the target compounds against selected tumor cell lines.

Series	Compd.	R	GI_50_ (µM) ^a^				Relative Resistance ^b^
			A549	DU145	KB	KBvin	
**I**	**10**	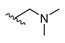	0.139	0.502	0.904	2.747	3.0
	**11**	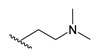	0.078	0.173	0.314	2.105	6.7
	**12**	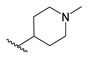	0.451	0.381	0.309	13.877	44.9
	**13**	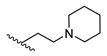	0.301	0.212	1.584	6.438	4.1
	**14**	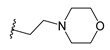	0.235	0.283	0.637	>10	-
	**15**	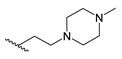	0.300	0.268	0.499	>10	-
	**16**	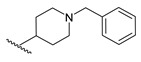	0.041	0.095	0.322	>10	-
	**17**	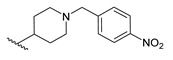	0.143	0.232	2.655	>10	-
	**18**	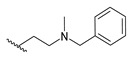	0.034	0.130	0.262	1.492	5.7
	**19**	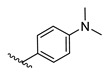	0.833	0.745	0.695	0.686	1.0
**II**	**20**	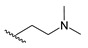	0.254	0.384	0.948	9.297	9.8
	**21**	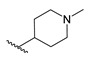	1.380	1.179	3.092	15.547	5.0
	**22**	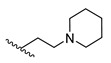	0.263	0.612	4.894	>10	-
	**23**	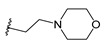	1.056	2.301	3.051	12.522	4.1
	**24**	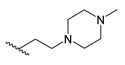	1.015	1.372	3.562	>10	-
	**25**	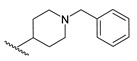	0.198	0.901	1.338	1.914	1.4
	**26**	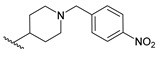	0.424	0.244	2.184	6.371	2.9
	**27**	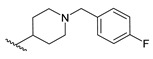	0.067	0.181	0.424	5.821	13.7
	**28**	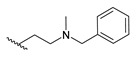	0.082	0.264	0.402	1.146	2.9
	**29**	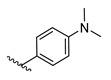	0.933	0.716	2.372	2.136	0.9
	**30**	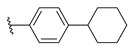	2.489	0.709	0.806	1.042	1.3
	**31**	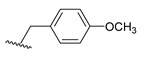	1.604	0.847	0.689	0.772	1.1
	**32**	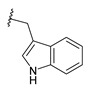	7.733	2.763	8.707	>10	-
	**33**	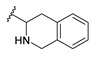	0.765	0.425	1.553	>10	-
	**34**	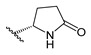	9.732	2.115	1.092	5.100	4.7
**-**	**6**	-	0.391	0.238	3.977	6.768	1.7
**-**	**2**	-	-	-	0.85	>10	>10

^a^ GI_50_, the concentration that causes 50% growth inhibition. A549: lung cancer, DU145: prostate cancer; KB: nasopharyngeal carcinoma; KBvin: vincristine resistant KB subline. ^b^ Relative resistance values are the GI_50_ values against KBvin cells over those against KB cells.

**Table 2:** Induction of protein-linked DNA breaks (PLDB) by selected compounds.

Compd.	%PLDB Formation ^a^	Compd.	%PLDB Formation ^a^
**10**	54 ± 12	**27**	285 ± 30
**11**	246 ± 28	**28**	273 ± 22
**18**	284 ± 38	**29**	33 ± 5
**19**	79 ± 18	**30**	64 ± 13
**25**	51 ± 9	**31**	82 ± 17
**6**	232 ± 31		

^a^ %PLDB formation was determined for compounds compared side-by-side in two independent experiments using the SDS/potassium precipitation method (please refer to [11]). Percent values represented mean levels ± SD of protein-linked DNA breaks induced by 5 µg/mL of test compound relative to the control (**2**) set arbitrarily as 100%.

**Table 3:** Inhibitory effects of compounds **2**, **6** and **28** against a panel of tumor cells.

Cell Lines ^a^	IC_50_ (µM) ^b^		
	2	6	28
Bel7402	>20	17.19 ± 9.49	4.09 ± 0.60
Bel7402/5-FU	>20	>20	5.05 ± 2.52
MCF-7	17.36 ± 4.66	7.21 ± 1.97	6.70 ± 2.30
MCF-7/DOX	>20	>20	>20
HCT116	9.67 ± 3.96	4.01 ± 1.34	3.71 ± 1.46
BGC823	>20	>20	10.34 ± 4.30
HepG2	3.88 ± 1.73	1.22 ± 1.40	2.18 ± 1.27
HeLa	9.83 ± 2.12	2.22 ± 0.45	2.87 ± 0.35

^a^ Bel7402: human hepatocellular carcinoma; Bel7402/5-FU: 5-fluorouracil resistant Bel7402 subline; MCF-7: human breast cancer; MCF-7/DOX: doxorubicin resistant MCF-7 subline; HCT116: human colon cancer; BGC823: human gastric adenocarcinoma; HepG2: human hepatocellular liver carcinoma; HeLa: human cervical cancer. ^b^ The IC_50_ values were represented as mean ± SD from three independent measurements.

**Table 4:** In vivo antitumor efficacy of compound **28** in hepatoma 22 (H22) mouse model.

Group	Dose (mg/kg × days)	Tumor Weight (g) ^b^	TGI (%) ^c^	Body Weight (g)	
				Day 0	Day 12
**Con ^a^**	-	1.70 ± 0.74	**-**	23.40 ± 0.97	31.19 ± 4.86
**28**	8 × 11	0.83 ± 0.50 *	**51.0**	23.50 ± 0.85	28.62 ± 2.57
**2**	26 × 8	0.42 ± 0.26 ***	**75.1**	23.60 ± 0.70	20.73 ± 3.97 ***

^a^ Con: vehicle control with sterile normal saline. ^b^ Both the tumor weight and the body weight were represented as mean ± SD (*n* = 10, * *p* < 0.05, *** *p* < 0.001 vs. Con). ^c^ TGI: tumor growth inhibition, which is a percentage calculated as (tumor weight of the control group—tumor weight of the treated group)/tumor weight of the control group.

## Data Availability

Not applicable.

## Sample Availability

Samples of compounds **10**–**34** are available from the authors.
